# A Well-Defined {[(PhCH_2_O)_2_P(CH_3_)_2_CHNCH(CH_3_)_2_]_2_PdCl_2_} Complex Catalyzed Hiyama Coupling of Aryl Bromides with Arylsilanes

**DOI:** 10.3390/molecules21080987

**Published:** 2016-07-29

**Authors:** Mengping Guo, Leiqing Fu, Jiamin Li, Lanjiang Zhou, Yanping Kang

**Affiliations:** 1 Institute of Coordination Catalysis, College of Chemistry and Bio-Engineering, Yichun University, Yichun 336000, China; fuleiqing2009@163.com (L.F.); lijiamin@163.com (J.L.); zhoulanjiang@163.com (L.Z.); kangyanping@163.com (Y.K.); 2 Engineering Center of Jiangxi University for Lithium Energy, Yichun University, Yichun 336000, China

**Keywords:** palladium (II) complex, Hiyama cross-coupling reaction, unsymmetrical biaryls, synthetic method

## Abstract

A palladium (II) complex {[(PhCH_2_O)_2_P(CH_3_)_2_CHNCH(CH_3_)_2_]_2_PdCl_2_} catalyzed Hiyama cross-coupling reaction between aryl bromides and arylsilanes has been developed. The substituted biaryls were produced in moderate to high yields, regardless of electron-withdrawing or electron-donating.

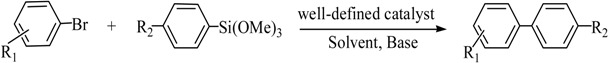

## 1. Introduction

**Figure 1:**
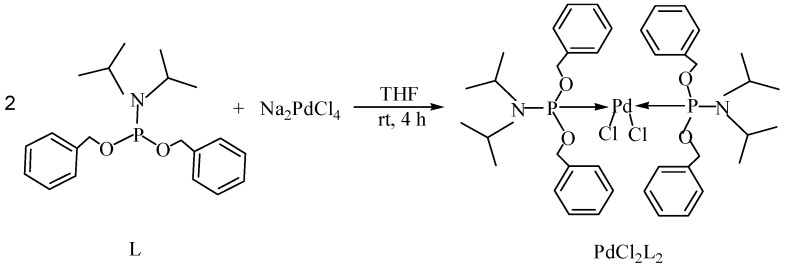
Synthesis of catalyst.

Transition metal-mediated cross-coupling reactions is one of the most powerful and versatile methods for the generation of unsymmetrical biaryls which are widely found as important structural units in pharmaceuticals [1], natural products [2], bioactive products [3], herbicides [4], conducting polymers [5], liquid crystal materials [6] and microelectrode arrays [7]. These cross-coupling transformations include organozinc (Negishi) [8] reaction, organotin (Stille) [9] reaction, organoboron (Suzuki-Miyaura) [10] reaction, and organomagnesium (Kumada) reaction [11]. Aryl halides have been widely used for a variety of cross-coupling reactions to form C-C bond. Recently, “comparatively unreactive” organosilane (Hiyama) reagents have been proposed as potential coupling partners due to their low cost and toxicity [12,13] ease of handling and stability in various chemical media [14]. Generally, great successes have been obtained using in situ catalytic systems as catalysts for Hiyama cross-coupling reactions [15,16,17,18]. However, the use of well-defined catalysts is still rare [14,19,20,21,22]. Herein, we have synthesized an air- and moisture-stable Pd (II) complex [(PhCH_2_O)_2_P(CH_3_)_2_CHNCH(CH_3_)_2_]_2_PdCl_2_ (PdCl_2_L_2_) containing electron-rich, sterically bulky phosphane (L), and it has been proved to be a highly efficient catalyst for Suzuki reaction with low Pd-catalyst loading (0.01%) [23]. Herein, this catalyst (Figure 1) is used for palladium-catalyzed Hiyama coupling reactions of arylsilanes with aryl bromides under ambient atmosphere.

## 2. Results and Discussion

### 2.1. Optimization of the Hiyama Reaction Condition

The catalyst PdCl_2_L_2_, which was inert to air and moisture, was composed of Na_2_PdCl_4_ with 2 equiv of the ligand L in THF at room temperature (Figure 1). The X-ray crystal structure of PdCl_2_L_2_ was shown as reported in [23]. The model reaction of bromobenzene with phenyltrimethoxysilane was initiated to optimize the cross-coupling conditions. Solvents used in the reaction were environmentally friendly and cheap and it avoid the troublesome solvents (NMP, DMF, etc.) that were conventionally applied in similar Hiyama reactions [14]. Results showed that no cross-coupled product was obtained when only H_2_O was used as solvent (Table 1, entry 1). However, it was exciting that a low yield (36%) was obtained in PEG solvent (Table 1, entry 2). Inspired by this, we explored different proportions of PEG and H_2_O (Table 1, entries 3–5), and it was discovered that PEG:H_2_O = 1:1 (volume ratio) was the efficient reaction system in this reaction (Table 1, entry 4) while the mixed solvent CH_3_CH_2_OH and H_2_O was not suitable for this reaction (Table 1, entry 6). Consequently, PEG:H_2_O = 1:1 (volume ratio) was chosen as the best solvent. To the best our knowledge, the base TBAF•3H_2_O played a key role in this reaction because TBAF•3H_2_O may show the function of the activation of arylsilane [24]. In order to avoid the use of TBAF•3H_2_O, we started to investigate a variety of inorganic salt base which should show the capability to favor the removal of silicon groups. Intriguingly, it gave a high yield of biphenyls (85%) when NaOH was applied as the base (Table 1, entry 7). Furthermore, the reaction did not proceed well when carried out with other base such as KOH, Et_3_N, Na_2_CO_3_, K_3_PO_4_ and NaOAc•3H_2_O (Table 1, entries 8–12). According to the above results, PEG:H_2_O = 1:1 (volume ratio), as the solvent, and NaOH as the base gave the best results. Since this catalytic system was not sensitive to oxygen, the reactions were carried out under air atmosphere without the protection of nitrogen.

### 2.2. Scope of the Substrates

The optimized reaction conditions were used in the Hiyama coupling reaction of various aryl bromides and arylsilanes with PdCl_2_L_2_ as a catalyst. The results were shown in Table 2. As expected, activated aryl bromide was smoothly converted into the corresponding products in 92% yields and 85% yields (Table 2, entries 2, 5). However, the electron donating group of aryl bromide would slightly decrease the reaction efficiency (Table 2, entries 3, 6). We also examined the electron donating group of arylsilane base on the resulting yields of the reactions. Electron donating group of arylsilane could be afforded biphenyl compounds at a higher temperature for 100 °C (Table 2, entries 4–8) but with lower yields. If aryl bromide and arylsilane both contained an electron donating group, the biphenyl yield clearly declined (Table 2, entry 6). Importantly, 1-bromonaphthalene was also applicable to these reaction conditions in moderate to good yield (Table 2, entry 7). The reaction system was also sufficiently stable for halogenated heterocyclic, so that 2-bromothiophene could be coupled with good efficiency (Table 2, entry 8).

## 3. Experimental Section

### 3.1. Reagents and Equipment 

NMR spectra were recorded using 400 MHz in DMSO-*d*_6_ solutions at room temperature (tetramethylsilane (TMS) was used as an internal standard) on a Bruker Avance III spectrometer (Billerica, MA, USA, see Supplementary Materials). All chemicals employed in the reaction were analytical grade, obtained commercially from Aldrich or Alfa Aesar and were used as received without any prior purification.

### 3.2. Synthesis of the Catalyst

The palladium complex PdCl_2_L_2_ was prepared using a method previously reported elsewhere [21]. A solution of 1 mmol (0.345 g) was added dropwise to a suspension of 0.5 mmol Na_2_PdCl_4_ (0.147 g) in THF (20.0 mL) and the reaction mixture was stirred at ambient temperature for 4 h (Figure 1). The volume was reduced to 5.0 mL and diethyl ether was added to precipitate a yellow powder which was then filtered off and washed with diethyl ether. The complex PdCl_2_L_2_ was obtained in 92% yield.

### 3.3. General Procedure for the Synthesis

A mixture of aryl bromide (1.0 mmol), arylsilane (1.2 mmol), NaOH (3.0 mmol), 4.0 mL solvent, PEG:H_2_O = 1:1 (volume ratio) and catalyst (0.02 mmol) was stirred at 80–100 °C for 2 h under air. The reaction was quenched with brine (15 mL) and extracted three times with ethyl acetate (3 × 10 mL). The organic phase was dried with MgSO_4_ for 4 h, filtered and concentrated under reduced pressure using a rotary evaporator. The crude products were re-crystallized by dichloromethane (2 mL) at −10 °C for 24 h. Filtered and dried, the purified products were identified by ^1^H-NMR and ^13^C-NMR spectroscopy.

### 3.4. Analytical Data of Representative Products

Biphenyl Yield 88%: mp: 70–71 °C; ^1^H-NMR (DMSO-*d*_6_): δ 7.66 (d, *J* = 7.2 Hz, 4H), 7.47 (d, *J* = 7.6 Hz, 4H), 7.37 (t, *J* = 7.2 Hz, 2H). ^13^C-NMR (DMSO-*d*_6_): δ 127.1, 127.8, 129.3, 140.7.

4-Acetyl-4′-methoxybiphenyl Yield 85%: mp: 153–154 °C; ^1^H-NMR (DMSO-*d*_6_): δ = 7.99 (d, *J* = 8.0 Hz, 2H), 7.79(d, *J* = 7.5 Hz, 2H), 7.77 (d, *J* *=* 8.0 Hz, 2H), 7.05 (d, *J* = 7.5 Hz, 2H), 3.81 (s, 3H), 2.59 (s, 3H). ^13^C-NMR (DMSO-*d*_6_): δ 27.1, 55.7, 115.0, 126.6, 128.6, 129.3, 131.5, 135.4, 144.6, 160.1, 197.8.

4-Methoxybiphenyl Yield 74% and Yield 72%: mp: 90 °C; ^1^H-NMR (DMSO-*d*_6_): δ = 7.61 (m, 4H), 7.43 (m, 2H), 7.32 (d, *J* = 7.2 Hz, 1H), 7.01 (d, *J* = 8.2 Hz, 2H), 3.79 (s, 3H, CH_3_). ^13^C-NMR (DMSO-*d*_6_): δ 55.6, 114.8, 126.6, 127.1, 128.2, 129.3, 133.0, 140.3, 159.3.

4-Acetylbiphenyl Yield 92%: mp: 121 °C; ^1^H-NMR (DMSO-*d*_6_): δ = 8.04 (d, *J* = 8.0 Hz, 2H), 7.82 (d, *J* = 8.0 Hz, 2H), 7.75 (d, *J* = 8.0 Hz, 2H), 7.51 (m, 1H), 7.44 (d, *J* *=* 8.0 Hz, 2H), 2.61 (s, 3H). ^13^C-NMR (DMSO-*d*_6_): δ 27.2, 127.3, 127.4, 128.8, 129.3, 129.5, 136.1, 139.3, 145.0, 197.9.

## 4. Conclusions

In conclusion, complex PdCl_2_L_2_ was demonstrated to be a highly active catalyst for the Hiyama coupling reaction of a range of aryl bromides with arylsilanes, affording the coupling products with moderate to high yields. This method is consistent with the concept of green chemistry, and further studies on the applicability of this catalyst system in other coupling reactions such as Sonogashira and amination are currently under investigation in our laboratory.

## Figures and Tables

**Table 1:** Optimization of the Hiyama reaction condition ^a^. 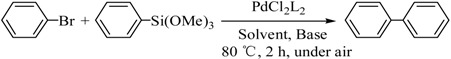

Entry	Solvent	Base	Yield (%) ^b^
1	H_2_O	K_2_CO_3_	0
2	PEG	K_2_CO_3_	36
3	PEG:H_2_O = 1:3	K_2_CO_3_	50
4	PEG:H_2_O = 1:1	K_2_CO_3_	73
5	PEG:H_2_O = 3:1	K_2_CO_3_	64
6	CH_3_CH_2_OH:H_2_O = 1:1	K_2_CO_3_	trace
7	PEG:H_2_O = 1:1	NaOH	85
8	PEG:H_2_O = 1:1	KOH	75
9	PEG:H_2_O = 1:1	Et_3_N	58
10	PEG:H_2_O = 1:1	Na_2_CO_3_	71
11	PEG:H_2_O = 1:1	K_3_PO_4_	62
12	PEG:H_2_O = 1:1	NaOAc•3H_2_O	69

^a^ Reaction conditions:1.0 mmol bromobenzene, 1.2 mmol arylsilane, 0.02 mmol catalyst, 4.0 mL solvent, volume ratio, 3.0 mmol base, 80 °C under air. All the reactions were carried out for 2 h; ^b^ Isolated yield was based on the bromobenzene.

**Table 2:** Scope and Limitations of the Substrates ^a^. 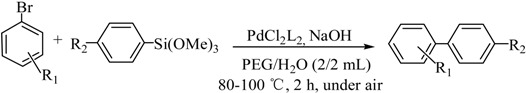

Entry	Ar-Br	Ar-Si(OMe)_3_	Product	Yield (%) ^b^
1	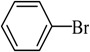	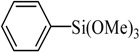	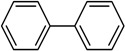	88
2	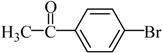	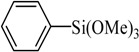	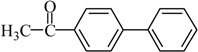	92
3	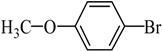	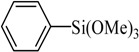	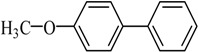	72
4 ^c^	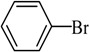	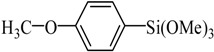	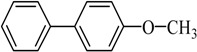	74
5 ^c^	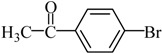	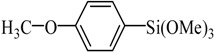	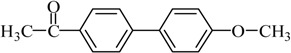	85
6 ^c^	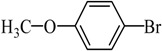	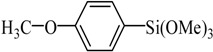	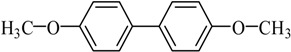	65
7 ^c^	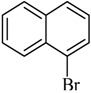	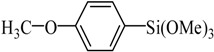	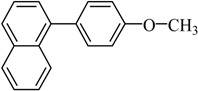	83
8 ^c^	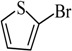	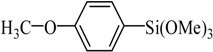	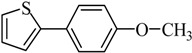	74

^a^ Reaction conditions:1.0 mmol aryl bromide, 1.2 mmol arylsilane, 0.02 mmol catalyst, 4.0 mL solvent, PEG:H_2_O = 1:1 (volume ratio), 3.0 mmol NaOH, 80 °C under air. All the reactions were carried out for 2 h; ^b^ Isolated yield was based on the aryl bromide; ^c^ The reaction temperature was 100 °C.

## Supplementary Materials

Supplementary materials can be accessed at: http://www.mdpi.com/1420-3049/21/8/987/s1.
