# Supplementary material for: A Well-Defined {[(PhCH2O)2P(CH3)2CHNCH(CH3)2]2PdCl2} Complex Catalyzed Hiyama Coupling of Aryl Bromides with Arylsilanes
Source: Molecules. 2016 Jul 29;21(8):987. doi: 10.3390/molecules21080987 (PMC6274572; doi:10.3390/molecules21080987)
Supplement: Supplementary file 1 [file molecules-21-00987-s001.pdf]

Supplementary Materials: A Well-Defined {[(PhCH2O)2P(CH3)2CHNCH(CH3)2]2PdCl2} Complex Catalyzed Hiyama Coupling of Aryl Bromides with Arylsilanes

Mengping Guo, Leiqing Fu, Jiamin Li, Lanjiang Zhou and Yanping Kang

NMR spectra was recorded on a Bruker Avance III (400 MHz) spectrometer using DMSO as the solvent.

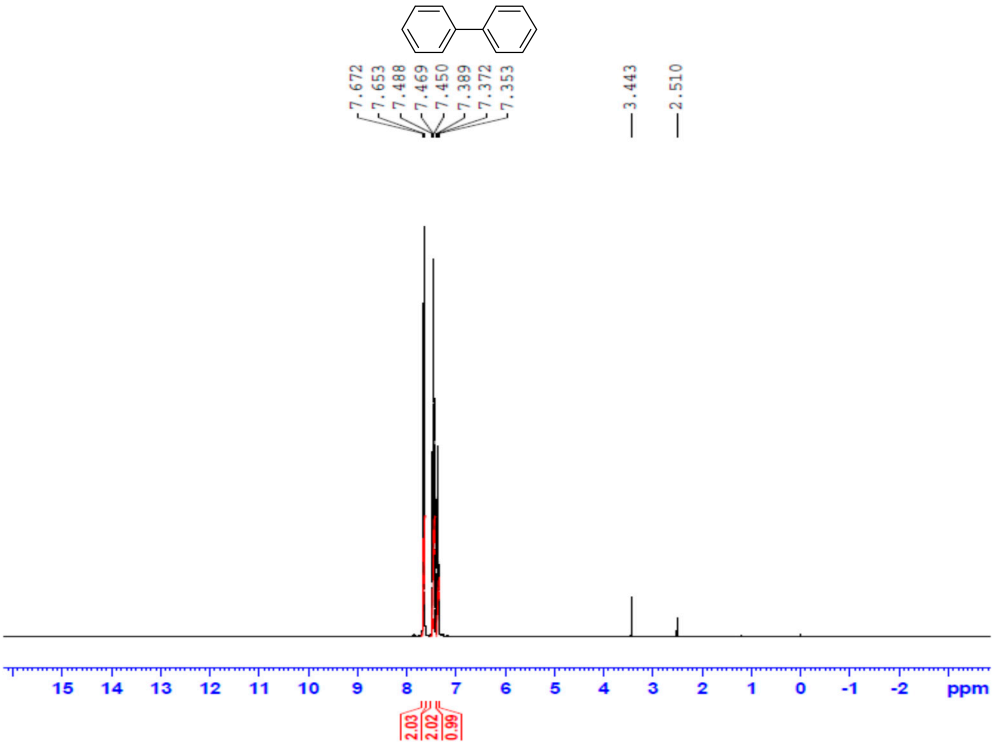

Figure S1. ^{1}H-NMR spectra of Biphenyl.

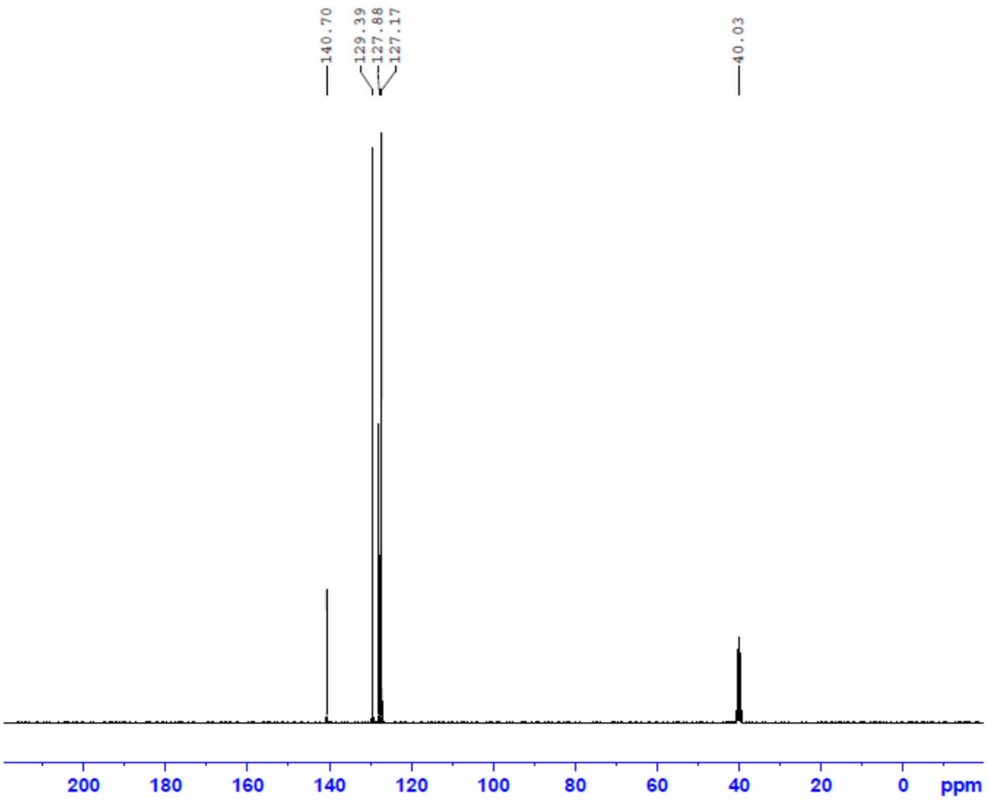

Figure S2. ^{13}C-NMR spectra of Biphenyl.

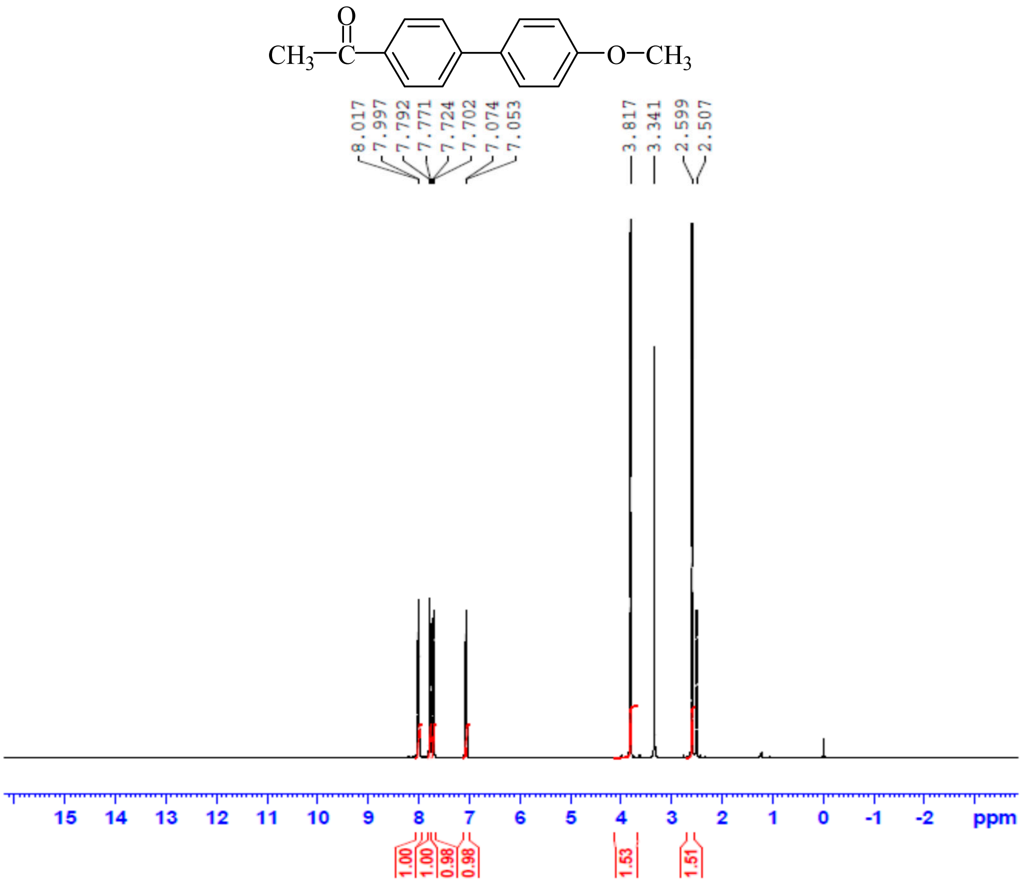

Figure S3. ^{1}H-NMR spectra of 4-Acetyl-4′-methoxybiphenyl.

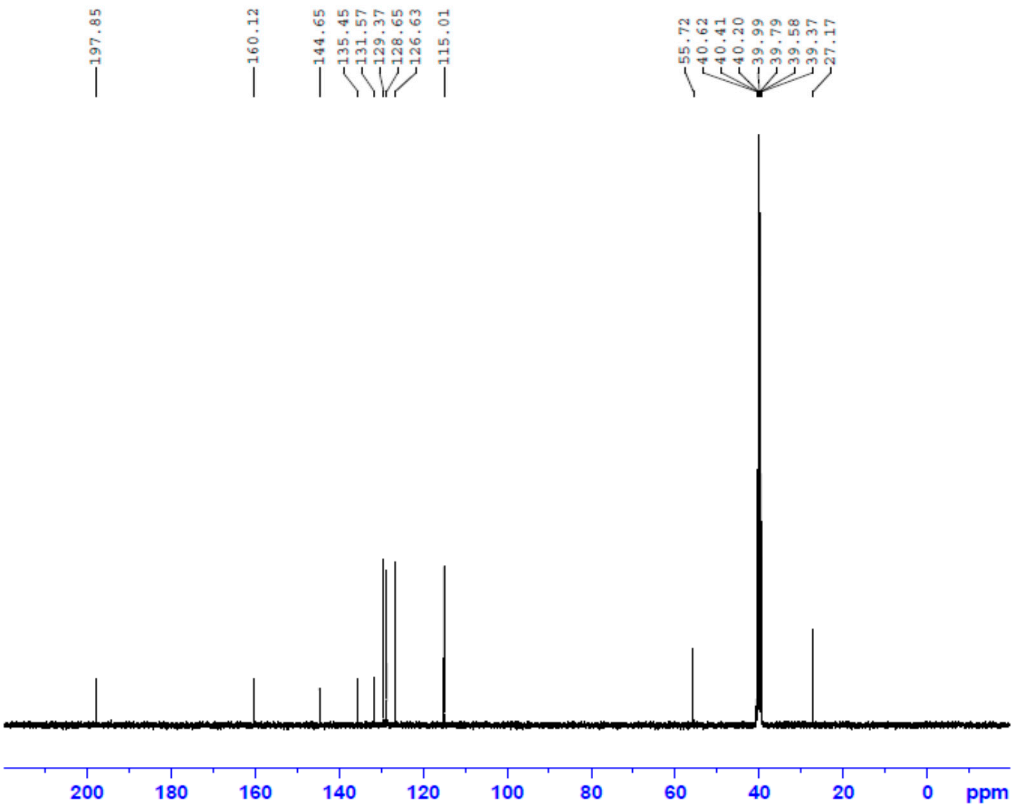

Figure S4. ^{13}C-NMR spectra of 4-Acetyl-4′-methoxybiphenyl.

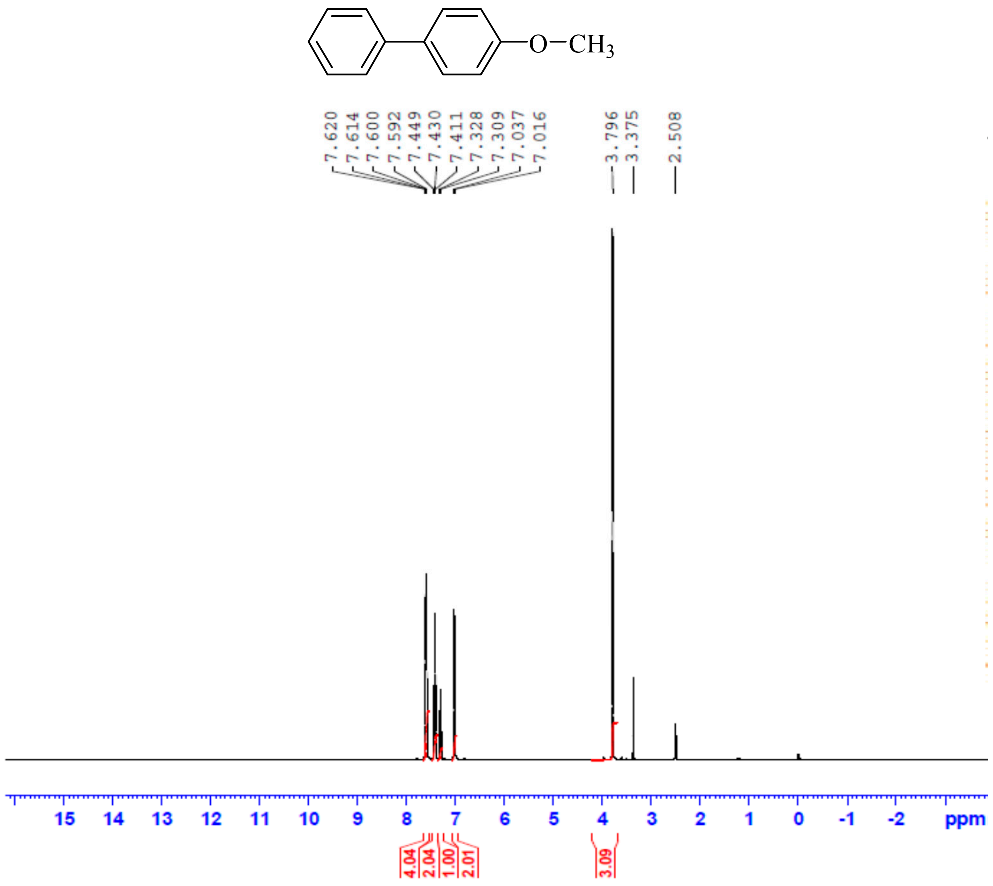

Figure S5. ^{1}H-NMR spectra of 4-Methoxybiphenyl.

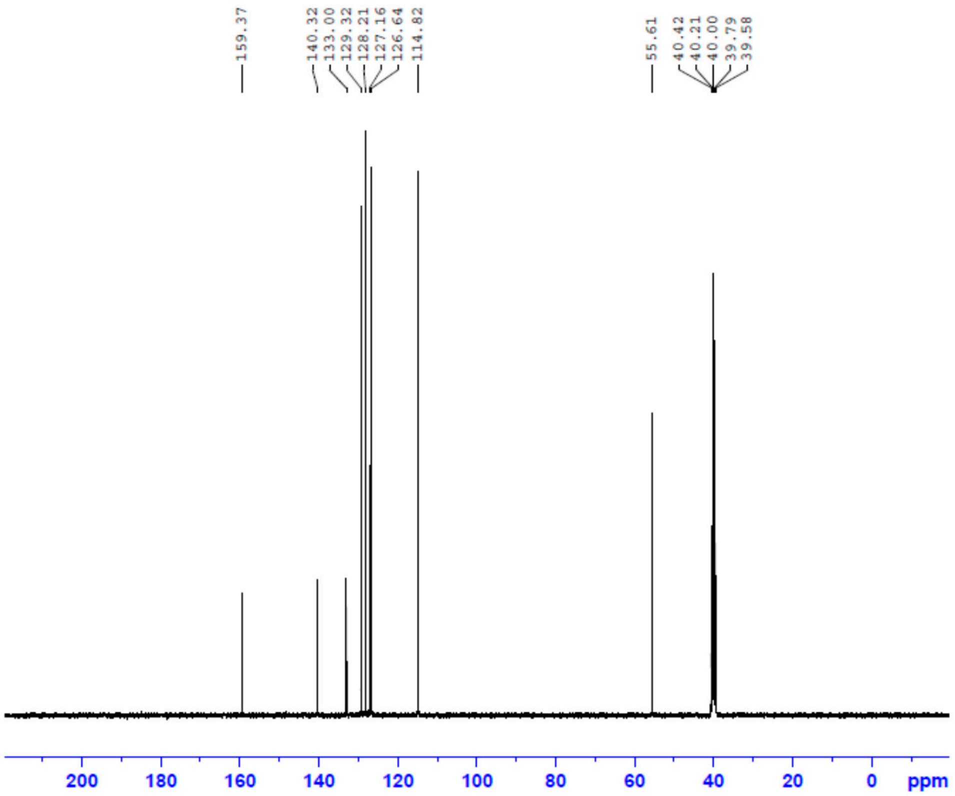

Figure S6. ^{13}C-NMR spectra of 4-Methoxybiphenyl.

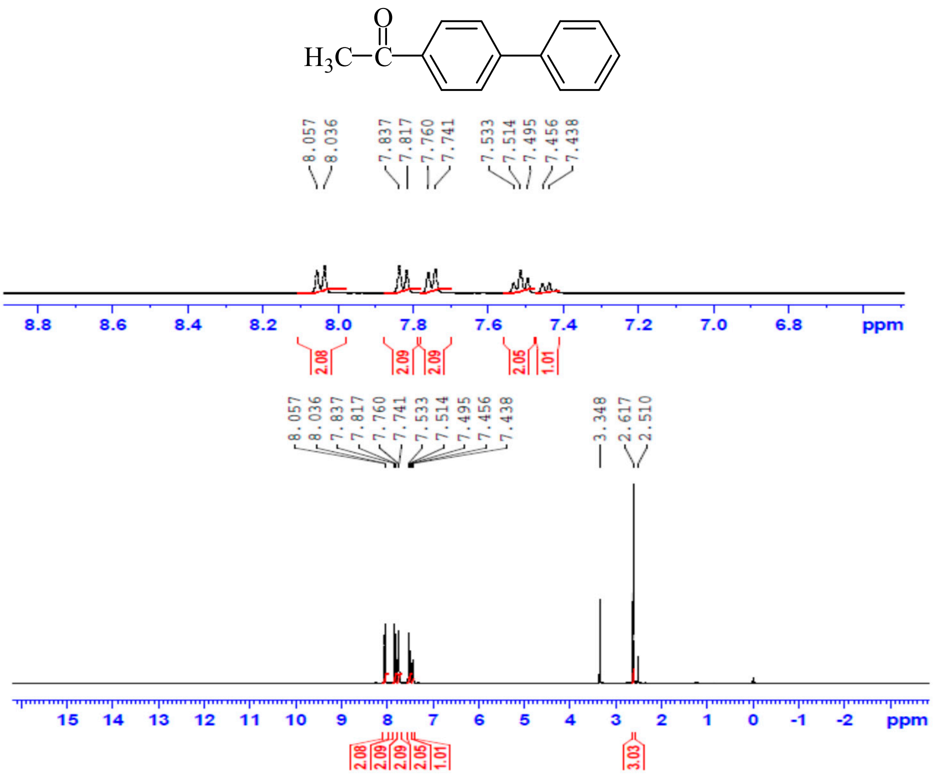

Figure S7. ^{1}H-NMR spectra of 4-Acetylbiphenyl.

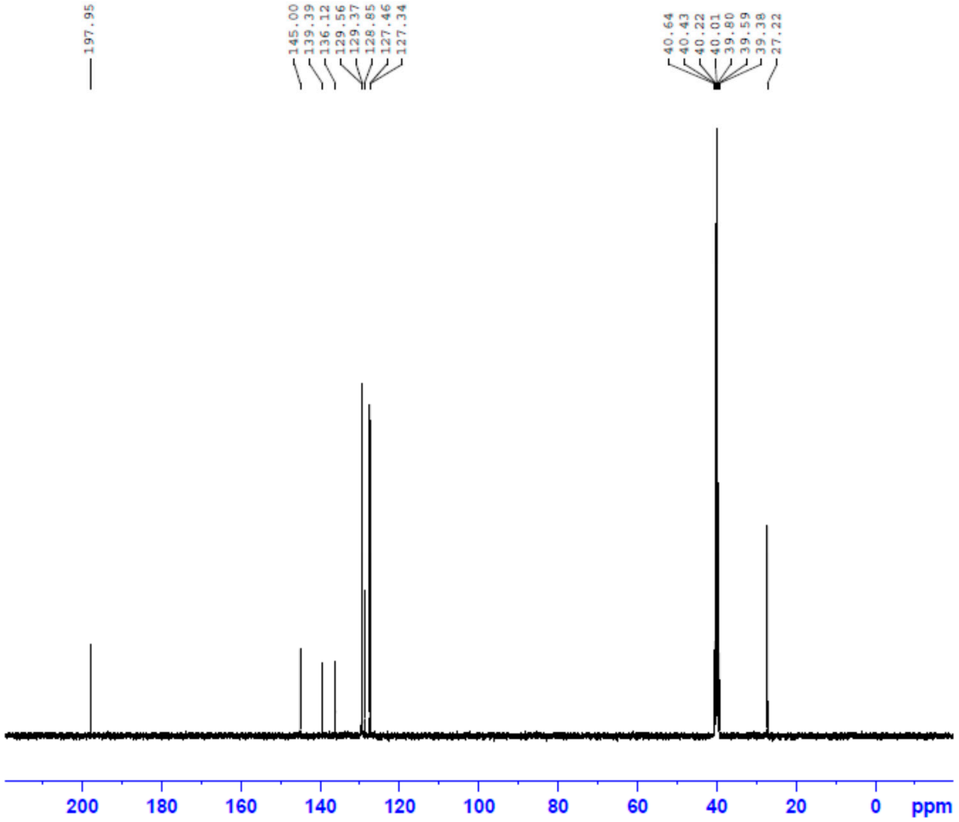

Figure S8. ^{13}C-NMR spectra of 4-Acetylbiphenyl.
